# Diffusion Is Directional: Innovative Diffusion Tensor Imaging to Improve Prostate Cancer Detection

**DOI:** 10.3390/diagnostics11030563

**Published:** 2021-03-20

**Authors:** Chen Shenhar, Hadassa Degani, Yaara Ber, Jack Baniel, Shlomit Tamir, Ofer Benjaminov, Philip Rosen, Edna Furman-Haran, David Margel

**Affiliations:** 1 Department of Urology, Rabin Medical Center, 39 Ze’ev Jabotinsky St, Petah Tikva 4941492, Israel; yaaraba1@clalit.org.il (Y.B.); jbaniel@clalit.org.il (J.B.); davidma4@clalit.org.il (D.M.); 2 Department of Biological Regulation, Weizmann Institute of Science, Rehovot 7610001, Israel; hadassa.degani@weizmann.ac.il; 3 Department of Imaging, Rabin Medical Center, 39 Ze’ev Jabotinsky St, Petah Tikva 4941492, Israel; shlomittamir10@gmail.com (S.T.); obenjaminov@szmc.org.il (O.B.); philipr@clalit.org.il (P.R.); 4 Department of Imaging, Shaare Zedek Medical Center, Jerusalem 9103102, Israel; 5 Life Sciences Core Facilities, Weizmann Institute of Science, Rehovot 7610001, Israel; Edna.Haran@weizmann.ac.il

**Keywords:** diffusion tensor imaging, magnetic resonance imaging, prostatic neoplasms, prostate cancer

## Abstract

In the prostate, water diffusion is faster when moving parallel to duct and gland walls than when moving perpendicular to them, but these data are not currently utilized in multiparametric magnetic resonance imaging (mpMRI) for prostate cancer (PCa) detection. Diffusion tensor imaging (DTI) can quantify the directional diffusion of water in tissue and is applied in brain and breast imaging. Our aim was to determine whether DTI may improve PCa detection. We scanned patients undergoing mpMRI for suspected PCa with a DTI sequence. We calculated diffusion metrics from DTI and diffusion weighted imaging (DWI) for suspected lesions and normal-appearing prostate tissue, using specialized software for DTI analysis, and compared predictive values for PCa in targeted biopsies, performed when clinically indicated. DTI scans were performed on 78 patients, 42 underwent biopsy and 16 were diagnosed with PCa. The median age was 62 (IQR 54.4–68.4), and PSA 4.8 (IQR 1.3–10.7) ng/mL. DTI metrics distinguished PCa lesions from normal tissue. The prime diffusion coefficient (λ_1_) was lower in both peripheral-zone (*p* < 0.0001) and central-gland (*p* < 0.0001) cancers, compared to normal tissue. DTI had higher negative and positive predictive values than mpMRI to predict PCa (positive predictive value (PPV) 77.8% (58.6–97.0%), negative predictive value (NPV) 91.7% (80.6–100%) vs. PPV 46.7% (28.8–64.5%), NPV 83.3% (62.3–100%)). We conclude from this pilot study that DTI combined with T2-weighted imaging may have the potential to improve PCa detection without requiring contrast injection.

## 1. Introduction

Multiparametric magnetic resonance imaging (mpMRI), including anatomical, dynamic contrast enhanced (DCE) and diffusion-weighted imaging (DWI), is an effective tool for the detection of prostate cancer (PCa) with sensitivity and specificity both over 80% [1,2]. MpMRI decreases the number of unnecessary prostate biopsies and improves the detection of clinically significant PCa [3,4,5]. However, MRI is far from being ideal. Current mpMRI protocols are limited by interobserver discrepancies, false-positives affected by prevalent findings, such as hyperplasia or inflammation, false-negatives in ductal variant adenocarcinoma [6] and a tendency to underestimate tumor volume [7].

Diffusion weighted imaging (DWI) is an important component in prostate imaging reporting and data system ver.2 (PI-RADS2) [1,8]. DWI quantifies the average Brownian movement of water molecules in tissue, but not direction-dependent movement. The direction and rates of water diffusion are restricted by complex microstructural tissue-barriers, such as ducts, fibers, and cells. Thus, diffusion directionality and anisotropy measured by diffusion tensor imaging (DTI) may reflect the microanatomical tissue structure and its disruption.

DTI metrics have been shown to identify cancerous lesions in glandular organs, such as the breast and pancreas [9,10]. The theory behind DTI and initial processing algorithms was first described by Basser et al. in 1994 [11].

As far as we know, previous studies of the utility of DTI for PCa detection have mostly analyzed only a part of the existing DTI metrics, and showed inconclusive, yet encouraging, results [12,13,14,15,16,17,18,19,20,21,22]. A recent paper by Gholizadeh et al. suggested that a model combining multiple DTI parameters has improved sensitivity and specificity for PCa detection [23].

In this pilot study, we applied a new standardized DTI protocol and a proprietary software to automatically process diffusion images recorded in 32 directions. We compared DTI results to DWI and mpMRI used in the standard clinical setting for PCa detection.

## 2. Materials and Methods

The research protocol was approved by the Rabin Medical Center Institutional Research Ethics Committee (protocol code 0357-16-RMC, first approved on 19/6/2016), and signed informed consent was obtained from all subjects.

### 2.1. Patients

Eighty (80) consecutive patients, 40–80 years old, planning to undergo prostate MRI for suspected PCa were recruited. Indication for MRI was elevated prostate-specific antigen (PSA). Men under 50 were screened using PSA due to family history. Study inclusion criteria were males undergoing evaluation for suspected prostate cancer using a 3T MRI scanner, able to provide informed consent. Exclusion criteria were contraindications to MRI, such as metallic implants. We applied a DTI protocol in addition to the standard mpMRI protocol. We collected respective clinical and pathological data for each patient. 

### 2.2. mpMRI Acquisition

Standard clinical mpMRI scans were performed on a 3T-scanner (Ingenia, Philips Medical Systems, Best, The Netherlands) using 32-channel posterior and 32-channel anterior torso coils. The acquisition protocols included the following: 1. an axial T2-weighted turbo spin-echo sequence (TR/TE 5209/120 ms, slice width 3 mm with 0.3 mm gap, turbo factor 24, in-plane resolution 0.9 × 1.1 mm, two averages, scan time 2:01 min); 2. an axial diffusion-weighted sequence using a single-shot echo-planar sequence (TR/TE 4669/91 ms, slice width 3 mm with 0.3 mm gap, turbo factor 45, in-plane resolution 3.0 × 3.1 mm, five b-values (0, 100, 1000, 1500 and 2000 s/mm^2^), SENSE factor 2.0, SPAIR fat suppression, two averages, scan time 4:54 min); 3. a DCE axial 3D, T1-weighted single-shot turbo field echo sequence with gadolinium injection (Dotarem^®^ contrast, TR/TE = 3.29/1.5 ms, flip angle 10^0^, slice width 3.0/−1.5 mm turbo factor 31, SPAIR fat suppression, in-plane resolution 1.8 × 1.8 mm, two averages, one pre and nine post contrast acquisitions, scan time 4:49 min).

### 2.3. mpMRI Analysis

Acquired images were reviewed by two fellowship-trained radiologists (S.T, O.B,) specializing in pelvic and abdominal imaging and experienced in prostate MRI reading and reporting. Malignant potential of lesions was stratified according to PI-RADS2 [1,8]. Lesions detected by mpMRI were targeted for biopsy.

For comparison with DTI values, apparent diffusion coefficient (ADC) maps were also marked with regions of interest (ROIs) labeled according to the PI-RADS2 score, and the average ADC value was calculated for each ROI. This was performed retrospectively within the previously marked lesions.

### 2.4. DTI Acquisition

A 2D axial DTI scan was added to the mpMRI protocol and acquired on the same 3T scanner, prior to contrast injection. The DTI protocol used monopolar spin-echo, echo-planar imaging with diffusion gradients in 32 directions at b values of 0 and 600 s/mm^2^, TR/TE = 4163/70 ms, SENSE factor (in-plane) of 2.6, 26 axial slices, 3 mm thick with a 0.3 mm gap, an in-plane resolution of 1.43 × 1.43 mm, and fat-saturation. The scan time for DTI was 4:36 min. 

### 2.5. DTI Image Processing and Analysis

Data were analyzed using a dedicated DTI software, developed and supervised by H. Degani and her team in the Weizmann Institute of Science (currently assigned to DDE MRI Solution Ltd, Tel Aviv, Israel) [24]. This DTI software calculated a symmetric diffusion tensor in each voxel (three-dimensional pixel) of tissue [25]. For each voxel, the analysis produced three eigenvectors (directions) and their corresponding eigenvalues (diffusion rate in these directions)—the directional diffusion coefficients, λ_1_, λ_2_, and λ_3_, sorted from highest to lowest [11,24,25]. When all three coefficients are equal, the diffusion is considered isotropic, meaning equal diffusion in all directions. Differences between the three diffusion coefficients suggest anisotropy or directional diffusion.

The three diffusion coefficients served to calculate the mean diffusivity (Equation (1)), a measure of the diffusion averaged over all directions; two anisotropy metrics: maximal anisotropy (Equation (2)), the difference between the maximal and minimal diffusion coefficients; and fractional anisotropy (Equation (3)), which is a normalized measure of diffusion anisotropy, varying between 0 (fully isotropic diffusion, equal in all directions) to 1 (infinite anisotropy with diffusion in one direction, fully restricted in the other directions).

Equations:

(1) $$\mathrm{MD}=\left(\lambda_{1}+\lambda_{2}+\lambda_{3}\right)/3$$

(2) $$\mathrm{MA}=\lambda_{1}-\lambda_{3}$$

(3) $$\mathrm{FA}=\frac{\sqrt{3\left[{\left(\lambda_{1}-\langle \lambda \rangle \right)}^{2}+{\left(\lambda_{2}-\langle \lambda \rangle \right)}^{2}+{\left(\lambda_{3}-\langle \lambda \rangle \right)}^{2}\right]}}{\sqrt{2\left(\lambda_{1}^{2}+\lambda_{2}^{2}+\lambda_{3}^{2}\right)}}$$

The software generated color-coded maps for the six DTI metrics (λ_1_, λ_2_, λ_3_, MD, MA, and FA) [24,25]. The maps were aligned to each other and to the anatomical T2-weighted images on a single screen to aid with lesion location and delineation. A scientist with prior experience in prostate MRI and breast MRI including DTI, but no experience in prostate DTI (H.D), identified lesions suspected as malignant by their low directional diffusion coefficients and low intensity in T2-weighted images. This interpreter had no prior knowledge of mpMRI results, pathology, or patient characteristics. For comparison, DTI metrics were calculated for normal-appearing regions in the same prostate.

We used a 3-level Likert scale (normal-appearing, low-suspicion, or high-suspicion for cancer) for categorizing the DTI findings. The final quantitative evaluation of the average values of the six DTI metrics within the ROI (similar to the DWI & ADC data from the DWI modality) was performed after unblinding and compared to the histopathological findings.

Separate analyses were performed for the peripheral zone (PZ) and central gland (CG) due to the marked difference in diffusion metrics between these areas.

### 2.6. Pathology

Prostate biopsies were performed when indicated by clinical exam, serum markers and standard mpMRI. Prostate biopsies were guided by the fusion of mpMRI data registered to trans-rectal ultrasound imaging using the Navigo^®^ (UC-Care, Yokneam, Israel) system, with a combined targeted and systematic template.

For the purpose of statistical analysis, non-cancerous proliferative lesions, such as high-grade prostatic intraepithelial neoplasia (PIN) and atypical small acinar proliferation (ASAP), were considered normal prostate tissue.

### 2.7. Statistical Analysis

Statistical analysis was performed using SPSS version 20 (IBM, Armonk, New York, USA).

We employed a Student’s *t*-test to evaluate differences in clinical, DWI ADC, and DTI metrics between normal tissue and cancerous lesions, using Bonferroni correction for multiple comparisons. We used the 3-level Likert scale to calculate specificity, sensitivity, negative predictive value (NPV), and positive predictive value (PPV) and compared them to those derived from the standard PI-RADS2 mpMRI scores. We performed receiver operating characteristic (ROC) curve analyses for the various DTI metrics and for conventional DWI ADC and calculated the area under the curve (AUC).

## 3. Results

### 3.1. Patients

DTI scans were successfully performed on 78 patients; 42 of them underwent biopsy, and 16 were diagnosed with PCa: eight had grade group 1 PCa, five had grade group 2 cancer, one had grade group 4 cancer, and two had grade group 5 cancer. The median age of the patients undergoing biopsies was 61.7 years (inter-quartile range: 54.4–68.4), and the median PSA was 4.8 (inter-quartile range: 1.3–10.7) ng/mL (Table 1). We were able to analyze all data using the software.

### 3.2. DTI Metrics

**Figure 1:**
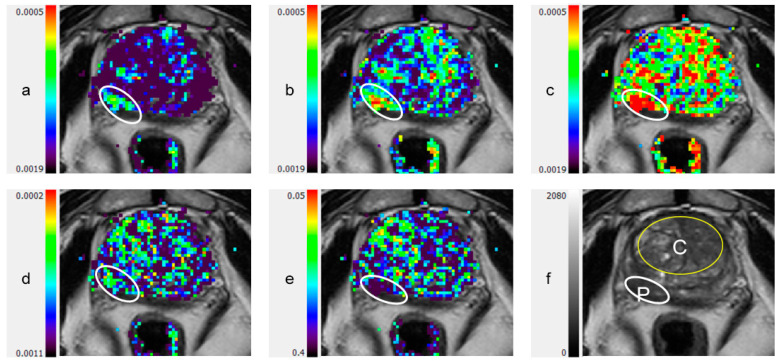
Peripheral zone lesion on color coded DTI Maps and T2-weighted image: (**a**) λ_1_; (**b**) λ_2_; (**c**) λ_3_; (**d**) maximal anisotropy (MA); (**e**) fractional anisotropy (FA); (**f**) T2-weighted image. C: central gland (transitional zone and central zone); DTI: diffusion tensor imaging; P: peripheral zone; White ellipsoid in (**a**) to (**e**): peripheral zone lesion.

**Figure 2:**
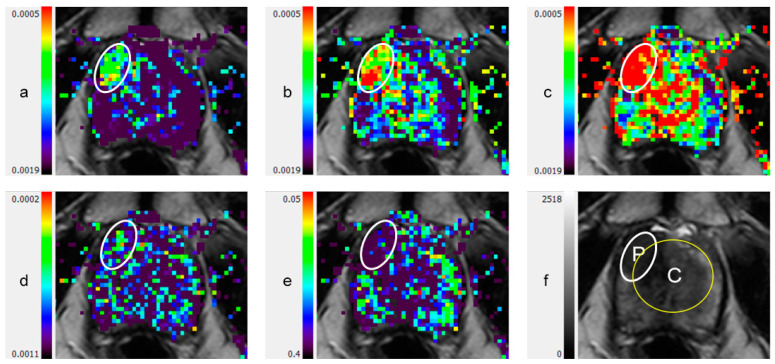
Anterior lesion involving peripheral zone and central gland, on color coded DTI maps and T2-weighted image: (**a**) λ_1_; (**b**): λ_2_; (**c**) λ_3_; (**d**) maximal anisotropy (MA); (**e**) fractional anisotropy (FA); (**f**) T2-weighted image. C: central gland (transitional zone and central zone); DTI: diffusion tensor imaging; P: peripheral zone; White ellipsoid in (**a**) to (**e**): anterior lesion.

Typical examples of DTI parametric maps of malignant lesions and the surrounding tissue in PZ and CG are shown in Figure 1 and Figure 2, respectively. In healthy subjects, the primary diffusion coefficient (λ_1_) values in the PZ and CG were (2.30 ± 0.32) × 10^−3^ mm^2^/s and (1.98 ± 0.11) × 10^−3^ mm^2^/s, respectively (mean ± SD, *p* = 0.002) (Table 2). 

In a post hoc analysis of the quantitative measurements of DTI and DWI metrics in lesions later determined to be malignant compared to normal tissue, the three diffusion coefficients, MD, and MA were reduced, with the best separation achieved with the primary diffusion coefficient, λ_1_. FA was slightly but significantly increased in PZ cancer, with a similar trend for cancer in the CG as well. Overall, the primary diffusion coefficient, λ_1_, was (2.30 ± 0.34) × 10^−3^ mm^2^/s (mean ± SD) in normal PZ and (1.6 ± 0.33) × 10^−3^ mm^2^/s in PZ cancer (*p* < 0.00001), and (2.00 ± 0.14) × 10^−3^ mm^2^/s in normal CG versus (1.3 ± 0.12) ×10^−3^ mm^2^/s in CG cancer (*p* < 0.00001). The values of secondary and tertiary diffusion coefficients (λ_2_, λ_3_), mean diffusivity (MD), and anisotropy indices in cancer tissue were also significantly different from those in the corresponding normal tissue (Table 3). Note that the values of λ_2_ and MD were equivalent, with less than 3% difference between these two metrics.

### 3.3. Diagnostic Accuracy

To compare the accuracy of DTI to conventional mpMRI, we used the 3-level Likert score (Table 4). First, we compared all suspicious DTI lesions (Likert score 2 and 3, Table 4a) to PI-RADS2 3-5 lesions (Table 4b). As shown in the 2 × 2 contingency tables, the DTI protocol doubled the specificity of MRI for PCa resulting in higher NPV and PPV. 

We performed the same comparison but limited suspicious lesions to those with the highest Likert score (i.e., 3, Table 4c) on DTI. We compared these lesions to those with PI-RADS2 4 and 5 scores (Table 4d). Similar to our initial findings, the DTI protocol was more sensitive and specific to identify highly suspicious lesions (Likert score 3) compared to conventional mpMRI.

### 3.4. ROC Curve Analysis

**Figure 3:**
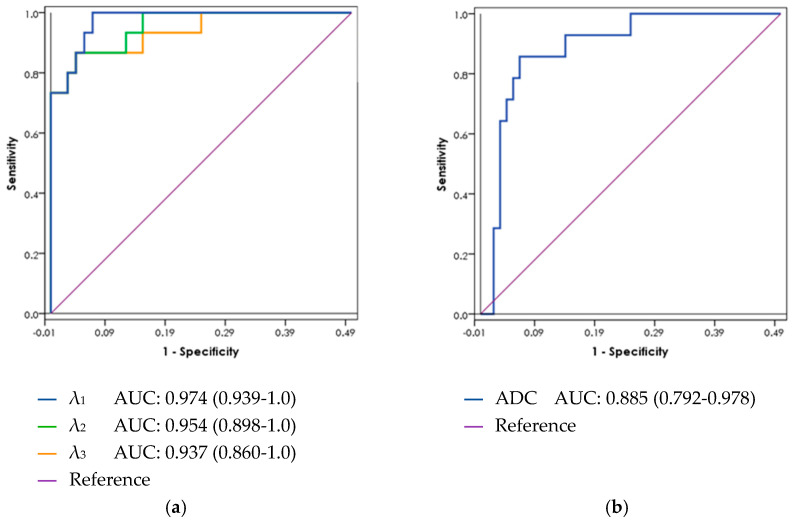
ROC curves for (**a**) DTI and (**b**) DWI metrics in the peripheral zones. ADC: apparent diffusion coefficient; AUC: area under the curve; DTI: diffusion tensor imaging; DWI: diffusion weighted imaging; PCa: prostate cancer; ROC: receiver operating characteristic.

In a receiver operating characteristic (ROC) curve analysis (Figure 3), the AUC for λ_1_ in the peripheral zone was 0.97 (95% confidence interval 0.94–1.0), with PCa threshold values below 2.1 × 10^−3^ mm^2^/s. For DWI ADC in the peripheral zone, the AUC was 0.89 (95% confidence interval 0.79–0.98) for values below 1.7 × 10^−3^ mm^2^/s as a threshold for PCa (Table 4). This difference did not reach statistical significance. ROC curve analysis was not performed for the CG due to the small number of central lesions found in this patient cohort.

## 4. Discussion

In this study, we demonstrated that an automated analysis of an adapted DTI protocol may improve the accuracy of PCa detection by MRI. We assessed DTI using two scores: a 3-level Likert scale and a quantitative measurement of diffusion metrics. Both scores exhibited good accuracy for PCa detection, with the Likert score showing a PPV of 78–93% (vs. 47–56% with ADC) and NPV of 89–92% (vs. 75–83% with ADC). The AUC calculated from the quantitative measurement reached 0.97 (95% CI 0.94–1.0) for the best-performing DTI value, λ_1_, vs. 0.89 (95% CI 0.79–0.98) for ADC values derived from DWI.

DWI measures the average water diffusion in tissue over three directions, and consistently shows a higher rate of diffusion in the PZ than in the CG [8], and a higher rate of diffusion in normal prostatic tissue than in PCa lesions. The prostate is composed of ductal and glandular elements within stromal tissue. The diffusion of water molecules is faster in the direction parallel to the walls of these microanatomical structures than in the directions perpendicular to them [24]. Thus, diffusion through the prostate has the property of direction in addition to its rate. This directionality, termed anisotropy, can be measured by DTI.

We know from similar studies on the breast that DTI can anatomically track the ductal tree [26] and detect breast cancer [24]. In the current study, we hypothesized that the same can be performed in the prostate, taking into consideration the directionality of glands and ducts in the peripheral, transitional, and central zones, as demonstrated in the classical work by McNeal [27].

Existing studies on DTI in the prostate generally use MRI manufacturer-supplied workstation DTI software [12,13,14,15,16,17,18,19,20,21], which gives ROI measurements of MD and FA, but the actual diffusion coefficients (λ_1_, λ_2_, and λ_3_) are not clearly shown. In this study, we applied diffusion gradients in 32 directions, and calculated the three directional diffusion coefficients (λ_1_, λ_2_, and λ_3_) and their derivatives (MD, MA, and FA). We were, therefore, able to study the detection efficiency of each DTI metric.

Previous studies show that ADC, or MD, is lower in PCa but disagree on FA. Some claim that FA values in PCa relative to normal prostate tissue are either higher [12,14,16,23], the same [28], or lower [29]. Uribe et al. conclude that any difference in FA values is attributable to a low signal to noise ratio and not to actual tissue differences [12].

From the existing literature, it appears that DTI has very little advantage over conventional DWI, since MD negates directionality and is generally equivalent to ADC from DWI, and FA results were, at best, inconclusive. However, we believe that MD and FA alone do not utilize the full potential of DTI. Our study shows that direct measurement of the primary diffusion coefficient, λ_1_, detected PCa better than any other DTI or DWI metrics. Moreover, measuring and displaying all DTI coefficient values in one screen with T2-weighted images enabled an interpreter inexperienced in prostate DTI to reach better PPV and NPV than experienced radiologists using formal mpMRI. One other study published to date has looked at more than the standard DTI parameters and found that a model combining diffusion coefficients and their 15 various products significantly increased the AUC for prostate cancer detection, when compared to DWI or only MD and FA from DTI [23]. This study was performed on post-biopsy DTI scans from 11 patients with high-risk PZ prostate cancer detected in a systematic biopsy, while our study looked at DTI and mpMRI scans performed before the prostate biopsy.

In our study, DTI performed better than DWI and mpMRI in detecting PCa lesions and in predicting both positive and negative biopsy results. This can either be due to the directionality of water diffusion, measured by DTI but not DWI, or due to the many repetitions of diffusion gradients (32) yielding a higher signal to noise ratio than the DWI protocol. The first theory is supported by the fact that λ_1_, a measurement of diffusion in the fastest direction, displayed better results than MD, which measures average diffusion and is comparable to ADC in DWI.

We successfully performed DTI scans on all patients and received meaningful data on all DTI measurements. The additional scan time of 4.6 min was in line with earlier published works [13,15,30,31]. This is an acceptable time which may further be lowered as we continue to define the characteristics of prostate DTI and more so if DTI is to replace DWI in the future. Furthermore, the DTI scan does not require contrast material injection. Since T2 and DTI appear to more efficiently detect PCa than mpMRI with DCE, perhaps in the future, DCE could be replaced by DTI, reducing both costs and patient contrast risks.

The strengths of this study are its prospective design and the comparison of DTI, DWI, PI-RADS2, and pathological findings. Another important feature of this study is the use of an adapted standard protocol and DTI analysis software developed specifically for the research of DTI measurements. In contrast, most other studies on prostate DTI have used manufacturer-supplied workstation DTI software with a limited set of output metrics [12,13,14,15,16]. The adaptability of the analysis software that we employed enabled us to find the specific metric that best describes directional (anisotropic) diffusion in the prostate.

The main study limitation was the small sample size. In addition, only patients with pathology were included in the analysis. It is possible that some patients with normal PSA and normal-appearing MRI had prostate cancer, limiting our ability to correctly estimate the false negative rate; however, we did not find it ethical to biopsy patients with negative MRI findings. 

## 5. Conclusions

This preliminary research showed the feasibility of DTI measurements of the prostate, suggesting that a specialized software assisted analysis of DTI metrics in combination with T2-weighted imaging may potentially improve PCa diagnosis without the need for contrast enhancement. Further validation studies are needed to verify the results of this pilot study.

## Figures and Tables

**Table 1:** Patient characteristics.

	All	NC	PCa	Significance *
	*n* = 42	*n* = 26	*n* = 16	
**Age,** median [IQR]	61.7 [54.4–68.4]	61.5 [51.1–65.4]	63.3 [55.5–71.0]	*p* = 0.21
**PSA** (ng/mL), median [IQR]	4.8 [1.3–10.7]	2.3 [1.0–6.8]	7.8 [4.2–11.2]	*p* = 0.29
**Prostate volume** by MRI (mL), median [IQR]	49.5 [34.8–70.0]	47.0 [33.2–69.0]	51.0 [35.0–91.0]	*p* = 0.26
**PSA density** (ng/mL/mL), median [IQR]	0.064 [0.035–0.158]	0.055 [0.026–0.125]	0.108 [0.056–0.218]	*p* = 0.11
**Suspicious DRE**	16%	16%	16%	*p* = 1
**Family History of prostate and related cancers**	26%	30%	19%	*p* = 0.39
**PCa: *n* (%)**				
Gleason 6 (3 + 3)			8 (50%)	
Gleason 7 (3 + 4)			5 (31%)	
Gleason 7 (4 + 3)			0	
Gleason 8 (4 + 4)			1 (6%)	
Gleason 9 (4 + 5)			2 (13%)	
Lesion in peripheral zone			11 (69%)	
Lesion in central gland			5 (31%)	

* For *t*-tests or chi-square tests comparing NC to PCa patients. DRE: digital rectal examination; IQR: inter-quartile range; NC: normal controls; PCa: prostate cancer; PSA: prostate specific antigen; SD: standard deviation.

**Table 2:** DTI and DWI metrics in normal prostate peripheral zone compared with central gland

Parameter		Peripheral Zone	Central Gland *	Mean Difference	2-Tailed Significance	Significance (Bonferroni-Corrected)
**DTI:**	**λ_1,_**	2.34 ± 0.25	2.03 ± 0.12	0.315	<0.0001	<0.0001
	**λ_2_**	1.87 ± 0.26	1.51 ± 0.13	0.357	<0.0001	<0.0001
	**λ_3_**	1.39 ± 0.30	1.00 ± 0.14	0.382	<0.0001	<0.0001
	**MD**	1.91 ± 0.27	1.52 ± 0.12	0.351	<0.0001	<0.0001
	**MA**	0.95 ± 0.11	1.01 ± 0.09	−0.067	0.007	0.15
	**FA**	0.27 ± 0.06	0.34 ± 0.04	−0.076	<0.0001	<0.0001
**DWI:**	**ADC**	2.33 ± 0.06	2.15 ± 0.03	0.177	0.076	1.0

* Central gland-transitional zone and central zone. Values are presented as mean ± standard deviation. Significance is corrected for multiple comparisons according to Bonferroni, across Table 2 and Table 3a,b. λ_1 to 3_, MD, MA, and ADC values presented in ×10^−3^ mm^2^/s. ADC: apparent diffusion coefficient; DTI: diffusion tensor imaging; DWI: diffusion weighted imaging; FA: fractional anisotropy; MA: maximal anisotropy; MD: mean diffusivity; PCa: prostate cancer.

**Table 3:** DTI and DWI metrics in normal and cancerous lesions, for (**a**) the peripheral zone; and (**b**) the central gland (transitional zone and central zone).

(**a**) Peripheral Zone						
**Parameter**		**Normal**	**PCa**	**Mean Difference**	**2-Tailed** **Significance**	**Significance** **(Bonferroni-Corrected)**
**DTI:**	**λ_1_**	2.34 ± 0.25	1.66 ± 0.30	0.684	<0.0001	<0.0001
	**λ_2_**	1.87 ± 0.26	1.23 ± 0.28	0.64	<0.0001	<0.0001
	**λ_3_**	1.39 ± 0.30	0.78 ± 0.26	0.61	<0.0001	<0.0001
	**MD**	1.91 ± 0.27	1.22 ± 0.28	0.644	<0.0001	<0.0001
	**MA**	0.95 ± 0.11	0.87 ± 0.10	0.074	0.027	0.57
	**FA**	0.27 ± 0.06	0.38 ± 0.06	−0.104	<0.0001	<0.0001
**DWI:**	**ADC**	2.33 ± 0.06	1.50 ± 0.04	0.083	<0.0001	<0.0001
(**b**) Central Gland						
**Parameter**		**Normal**	**PCa**	**Mean Difference**	**2-Tailed** **Significance**	**Significance** **(Bonferroni-Corrected)**
**DTI:**	**λ_1,_**	2.03 ± 0.12	1.26 ± 0.13	0.768	<0.0001	<0.0001
	**λ_2_**	1.51 ± 0.13	0.85 ± 0.05	0.663	<0.0001	<0.0001
	**λ_3_**	1.00 ± 0.14	0.46 ± 0.07	0.545	<0.0001	<0.0001
	**MD**	1.52 ± 0.12	0.85 ± 0.04	0.663	<0.0001	<0.0001
	**MA**	1.01 ± 0.09	0.80 ± 0.18	0.21	0.0003	0.0063
	**FA**	0.34 ± 0.04	0.45 ± 0.08	−0.111	0.078	1.0
**DWI:**	**ADC**	2.15 ± 0.03	1.35 ± 0.03	0.08	<0.0001	<0.0001

Values are presented as mean ± standard deviation. Significance is corrected for multiple comparisons according to Bonferroni, across Table 2 and Table 3a,b. λ_1 to 3_, MD, MA, and ADC values presented in ×10^−3^ mm^2^/s. ADC: apparent diffusion coefficient; DTI: diffusion tensor imaging; DWI: diffusion weighted imaging; FA: fractional anisotropy; MA: maximal anisotropy; MD: mean diffusivity; PCa: prostate cancer.

**Table 4:** 2 × 2 Contingency tables for diagnostic accuracy comparing conventional mpMRI to DTI. (**a**) DTI; (**b**) mpMRI; (**c**) DTI; (**d**) mpMRI.

**a. DTI** Likert score 2 and 3 were considered as DTI-positive						
	Pathology					
	Pos	Neg		sensitivity	87.5%	[71.3–100%]
DTI Pos	14	4	18	specificity	84.6%	[70.7–98.5%]
DTI Neg	2	22	24	PPV	77.8%	[58.6–97.0%]
	16	26	42	NPV	91.7%	[80.6–100%]
**b. mpMRI** PI-RADS2 3, 4 and 5 lesions on mpMRI were considered as mpMRI-positive						
	Pathology					
	Pos	Neg		sensitivity	87.5%	[71.3–100%]
mpMRI Pos	14	16	30	specificity	38.5%	[19.8–57.2%]
mpMRI Neg	2	10	12	PPV	46.7%	[28.8–64.5%]
	16	26	42	NPV	83.3%	[62.3–100%]
**c. DTI** Likert score 3 on DTI was considered as DTI-positive						
	Pathology					
	Pos	Neg		sensitivity	81.3%	[62.1–100%]
DTI Pos	13	1	14	specificity	96.2%	[88.8–100%]
DTI Neg	3	25	28	PPV	92.9%	[79.4–100%]
	16	26	42	NPV	89.3%	[77.8–100%]
**d. mpMRI** PI-RADS2 4 and 5 lesions on mpMRI were considered as mpMRI-positive						
	Pathology					
	Pos	Neg		sensitivity	62.5%	[38.8–86.2%]
mpMRI P	10	8	18	specificity	69.2%	[51.5–87.0%]
mpMRI N	6	18	24	PPV	55.6%	[32.6–78.5%]
	16	26	42	NPV	75.0%	[57.7–92.3%]

Pathology was determined by targeted MRI-TRUS fusion prostate biopsies. DTI: diffusion tensor imaging; mpMRI: multi-parametric magnetic resonance imaging, Neg: negative; NPV: negative predictive value; Pos: positive; PPV: positive predictive value; PI-RADS2: prostate imaging reporting and data system (version 2). Sensitivity, specificity, NPV, and PPV are given as a percentage (95% confidence interval).

## Data Availability

The data presented in this study are not publicly available due to patient privacy restrictions.
